# Advanced 3D visualisation of detailed neuronal models using the Open Source Brain repository and interaction with other neuroinformatics resources

**DOI:** 10.1186/1471-2202-14-S1-P363

**Published:** 2013-07-08

**Authors:** Padraig Gleeson, Matteo Cantarelli, Eugenio Piasini, R Angus Silver

**Affiliations:** 1 Department of Neuroscience, Physiology and Pharmacology, University College London, London, UK

## 

With the ever increasing complexity of models and applications used in computational neuroscience it is becoming clear that no one researcher or lab can produce and maintain detailed models which are applicable to a wide range of research questions, and can be used across the diverse tools available for simulation and analysis. A more collaborative approach to neuronal model development, inspired by the success of the open source software movement, is required [1]. The Open Source Brain repository (OSB, http://www.opensourcebrain.org) is being developed to enable researchers to collaboratively develop computational models of cells and networks which are well tested and documented and can be run across multiple simulators. This is enabled through the use of advanced distributed version control technologies and by converting the models to open, standardised formats like NeuroML [2], allowing the models to be used by the increasing range of simulators and other applications in the NeuroML "ecosystem". The current version of OSB contains models from diverse brain regions including neocortex and cerebellum, invertebrate models as well as more abstract cell and network models. OSB complements existing model archives which have traditionally concentrated on providing snapshots of published models. The cell, ion channel, synapse and network models in OSB develop over time to ensure they reflect known physiology and best practices in modelling. OSB users can comment on/extend/help to debug any model as well as contribute their own.

**Figure 1:**
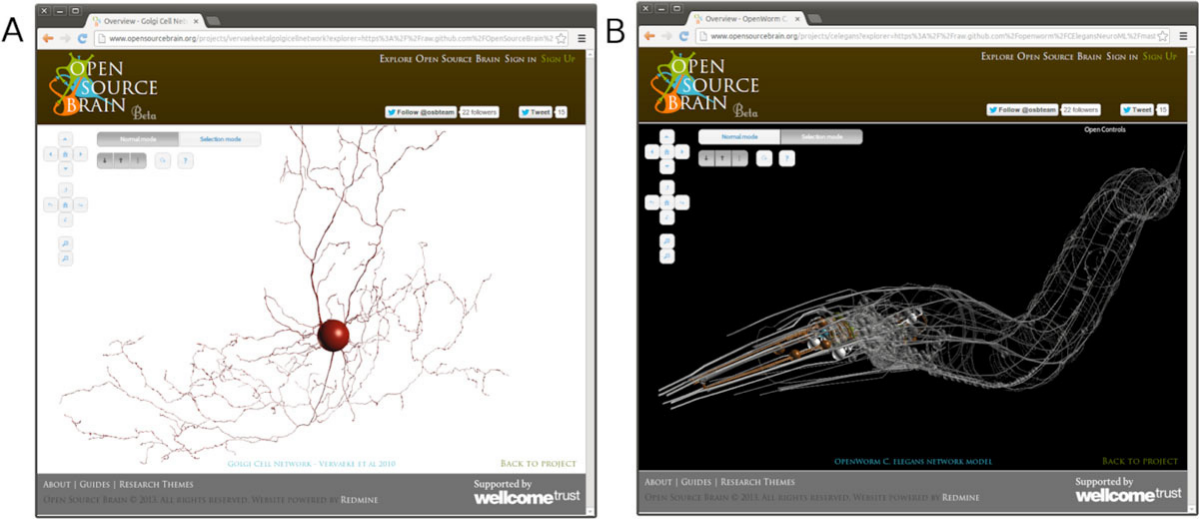
**Screenshots of OSB 3D Explorer**. A) Cerebellar Golgi cell. B) 302 neurons in OpenWorm C. elegans model

A major recent addition to the functionality of OSB is the 3D Explorer, allowing cells and networks in NeuroML format to be viewed in a browser (using WebGL, so requiring no additional software installation), and their structure and physiological properties examined (Figure 1). This means modellers need only commit models in NeuroML format to their project repositories (e.g. on GitHub) to have access to this advanced feature. In addition, we have created a number of "Technology Showcase" projects, with the aim of illustrating how OSB can interact with other specialist neuroinformatics resources and tools. These include NeuroMorpho, which provides neuronal reconstructions in multiple formats including NeuroML, NeuroElectro, providing structured data on electrophysiological properties of identified neurons which can be used to test the behaviour of OSB models, and Virtual Fly Brain, which provides detailed information on drosophila neurons and brain regions. Close links with other initiatives including ModelDB, NeuroLex and the OpenWorm project (http://openworm.org, Figure 1B) are also being developed. This work has been primarily funded by the Wellcome Trust (086699/095667).
